# Effect of Down syndrome and keratoconus on corneal density and volume: a triple comparative study

**DOI:** 10.1038/s41598-020-66108-4

**Published:** 2020-06-04

**Authors:** Soheila Asgari, Mohammadreza Aghamirsalim, Shiva Mehravaran, Hassan Hashemi

**Affiliations:** 1 Noor Ophthalmology Research Center, Noor Eye Hospital, Tehran, Iran; 2 Translational Ophthalmology Research Center, Tehran University of Medical Science, Tehran, Iran; 3 ASCEND Center for Biomedical Research, Morgan State University, Baltimore, MD USA

**Keywords:** Epidemiology, Preclinical research

## Abstract

Keratoconus (KCN) and Down syndrome affect the corneal density and volume. In this study included Down syndrome patients with and without KCN (24 Down-KCN and 204 Down-nonKCN eyes) and normal age- and gender-matched individuals (184 eyes). Studied parameters were the corneal density measured with Pentacam HR in 5 concentric zones and annuli (0–2 mm, 2–6 mm, 6–10 mm, 10–12 mm, and 0–12 mm) in 4 different depth layers (anterior 120 µm, posterior 60 µm, middle layer, and the full thickness of the cornea), and the 10 mm zone corneal volume. In Down-KCN, Down-nonKCN, and control groups, respectively, mean full thickness density in the 0–12 mm zone was 19.35 ± 2.92, 17.85 ± 2.55, and 15.78 ± 2.67 GSU, and mean corneal volume was 57.45 ± 4.37, 56.99 ± 3.46, and 61.43 ± 3.42mm^3^. All density readings were significantly different between the three studied groups (all P < 0.01) except full thickness density in 0–2 mm and 2–6 mm (P > 0.05) and corneal volume (P = 0.519) between Down-KCN and Down-nonKCN groups; these inter-group densitometry differences within the 6 mm zone were only in the middle layer, and not the anterior or posterior thickness layers (all P > 0.05). Corneal density increased with age and corneal thickness, but there was no significant relationship with gender. Overall, Down syndrome is associated with increased density and light scatter in all corneal layers up to the 12 mm diameter. In Down patients with KCN, the increased light scatter and density in the 6 mm zone is only in the middle thickness layer. Corneal volume is reduced in Down syndrome irrespective of the presence or absence of KCN.

## Introduction

Corneal density is an indicator of corneal transparency, and densitometry readings provide useful information about corneal clarity. Using the new Pentacam (Oculus Optikgeräte GmbH, Wetzlar, Germany) densitometry module, it is possible to evaluate backscattered light in different corneal regions expressed in standardized grayscale units (GSU) on a scale from 0 (for no clouding in the cornea) to 100 (maximum opacity and an opaque cornea)^1^. The range of corneal density has been described for healthy subjects^2^, keratoconus (KCN) patients^3,4^, and high myopic eyes^5^. Also, there have been studies reporting density changes after corneal cross-linking^6,7^, refractive surgeries^8,9^, and keratoplasty^10^. All these studies point to the value of this index in distinguishing normal from abnormal corneas and assessing the efficacy of different treatments.

Down syndrome patients have a different corneal structure compared to the normal population. Studies have shown that their corneas are steeper and thinner than normal^11–13^. These findings can be interpreted as KCN, especially early stage KCN, such that the reported prevalence of KCN in Down syndrome patients has been as high as 71.3%^11^. Therefore, it is necessary to properly define KCN diagnostic criteria and the normal range of common KCN indices for this population. One such index is the corneal density which can discriminate between KCN and normal eyes, [3, 4] but its normal range has not been determined in Down syndrome individuals. This study has been designed to assess corneal density in three groups (Down syndrome patients with and without KCN and a normal comparison group) and compare results to determine the individual effects of these conditions and answer the following questions: What is the effect of Down syndrome on corneal density and volume? How does KCN affect the corneal density and volume in Down syndrome patients? What is the normal range of corneal density and volume readings in this population?

## Results

Of the 250 participants with Down syndrome, 16 were excluded from the study due to concurrent autism or Klinefelter syndrome, and from the 200 subjects enrolled in the normal control group (non-Down and non-KCN), 10 were excluded due to a family history of mental disability. Corneal density and volume data were available for 228 of the 234 Down syndrome patients (24 Down-KCN and 204 Down-nonKCN) and 184 of the 190 subjects in the normal control group. Mean age in the Down-KCN, Down-nonKCN, and control groups was 16.71 ± 4.25, 17.19 ± 4.84, and 17.30 ± 4.47 years (P = 0.836), and 45.8%, 53.9%, and 50.5% of them were male (P = 0.666), respectively.

Table 1 compares the mean corneal density in the three anterior, middle, and posterior layers, and the corneal volume in the three groups. Most corneal density indices were significantly higher in Down-KCN than Down-nonKCN subjects; exceptions were anterior density in the 0–2 mm (P = 0.107), 2–6 mm (P = 0.171), and 10–12 mm annuli (P = 0.102), posterior density in the 0–2 mm (P = 0.111), and 2–6 mm annuli (P = 0.078), and the corneal volume (P = 0.519). All indices in the Down-KCN group were higher than normal (all P < 0.05). All indices in the Down-nonKCN group were higher than the normal control group except for anterior density in the 10–12 mm annulus (P = 0.654).

**Table 1:** Corneal density (standardized grayscale units) and volume (mm^3^) indices in Down patients with (Down-KCN) and without keratoconus (Down-nonKCN) and the normal age- and gender-matched control group.

Index	Annulus	Down-KCN (n = 24 eyes)	Down-nonKCN (n = 204 eyes)	Normal (n = 184 eyes)	P-value*	P-value**	P-value***
Density in anterior layer (120 µm)	0–2 mm	26.62 ± 4.75	25.16 ± 4.14	21.75 ± 4.47	0.116	<0.001	<0.001
	2–6 mm	23.57 ± 3.73	22.49 ± 3.67	19.71 ± 3.90	0.183	<0.001	<0.001
	6–10 mm	24.22 ± 5.88	21.51 ± 4.14	18.19 ± 3.73	0.002	<0.001	<0.001
	10–12 mm	34.33 ± 11.40	30.45 ± 10.88	30.04 ± 6.84	0.055	0.035	0.665
	0–12 mm	25.65 ± 4.46	23.76 ± 3.81	21.23 ± 3.90	0.025	<0.001	<0.001
Density in middle layer	0–2 mm	17.48 ± 3.06	16.32 ± 2.52	14.15 ± 2.42	0.033	<0.001	<0.001
	2–6 mm	15.53 ± 2.49	14.61 ± 2.27	12.87 ± 2.07	0.050	<0.001	<0.001
	6–10 mm	16.52 ± 3.40	14.67 ± 2.64	12.38 ± 2.09	0.002	<0.001	<0.001
	10–12 mm	25.32 ± 6.87	21.80 ± 6.56	19.03 ± 4.11	0.004	<0.001	<0.001
	0–12 mm	17.47 ± 2.71	15.99 ± 2.45	13.92 ± 2.21	0.004	<0.001	<0.001
Density in posterior layer (60 µm)	0–2 mm	14.64 ± 2.08	13.87 ± 1.73	12.34 ± 2.69	0.111	<0.001	<0.001
	2–6 mm	13.43 ± 1.76	12.69 ± 1.63	11.38 ± 2.28	0.078	<0.001	<0.001
	6–10 mm	14.69 ± 2.25	13.32 ± 2.06	11.59 ± 2.13	0.003	<0.001	<0.001
	10–12 mm	19.77 ± 4.75	17.47 ± 4.16	14.91 ± 3.15	0.005	<0.001	<0.001
	0–12 mm	14.91 ± 1.93	13.81 ± 1.81	12.18 ± 2.29	0.013	<0.001	<0.001
Corneal volume	10 mm	57.45 ± 4.37	56.99 ± 3.46	61.43 ± 3.42	0.519	<0.001	<0.001

*Comparison of Down-KCN and Down-nonKCN groups.

**Comparison of Down-KCN and normal groups.

***Comparison of Down-nonKCN and normal groups.

**Figure 1:**
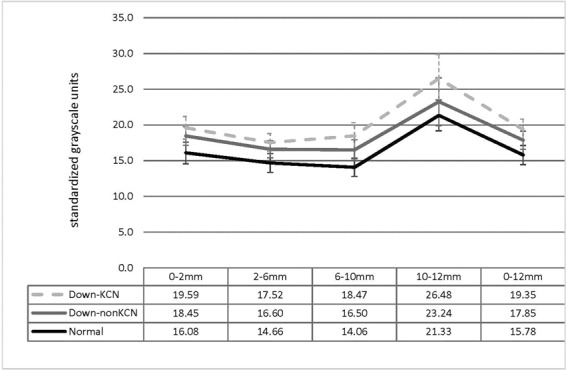
Full thickness corneal density (mean and standard deviation) in different zones and annuli centered on the apex in Down patients with (Down-KCN) and without keratoconus (Down-nonKCN) and the normal age- and gender-matched control group.

Figure 1 shows the full thickness density readings in different zones and annuli which were all significantly different between the three studied groups (all P < 0.01) except full thickness density in the 0–2 mm zone between Down-KCN and Down-nonKCN groups (19.59 ± 3.17 vs. 18.45 ± 2.64 GSU, respectively, P = 0.184), and the 2–6 mm annulus between Down-KCN and Down-nonKCN groups (17.52 ± 2.55 vs. 16.60 ± 2.41 GSU, respectively, P = 0.235).

**Figure 2:**
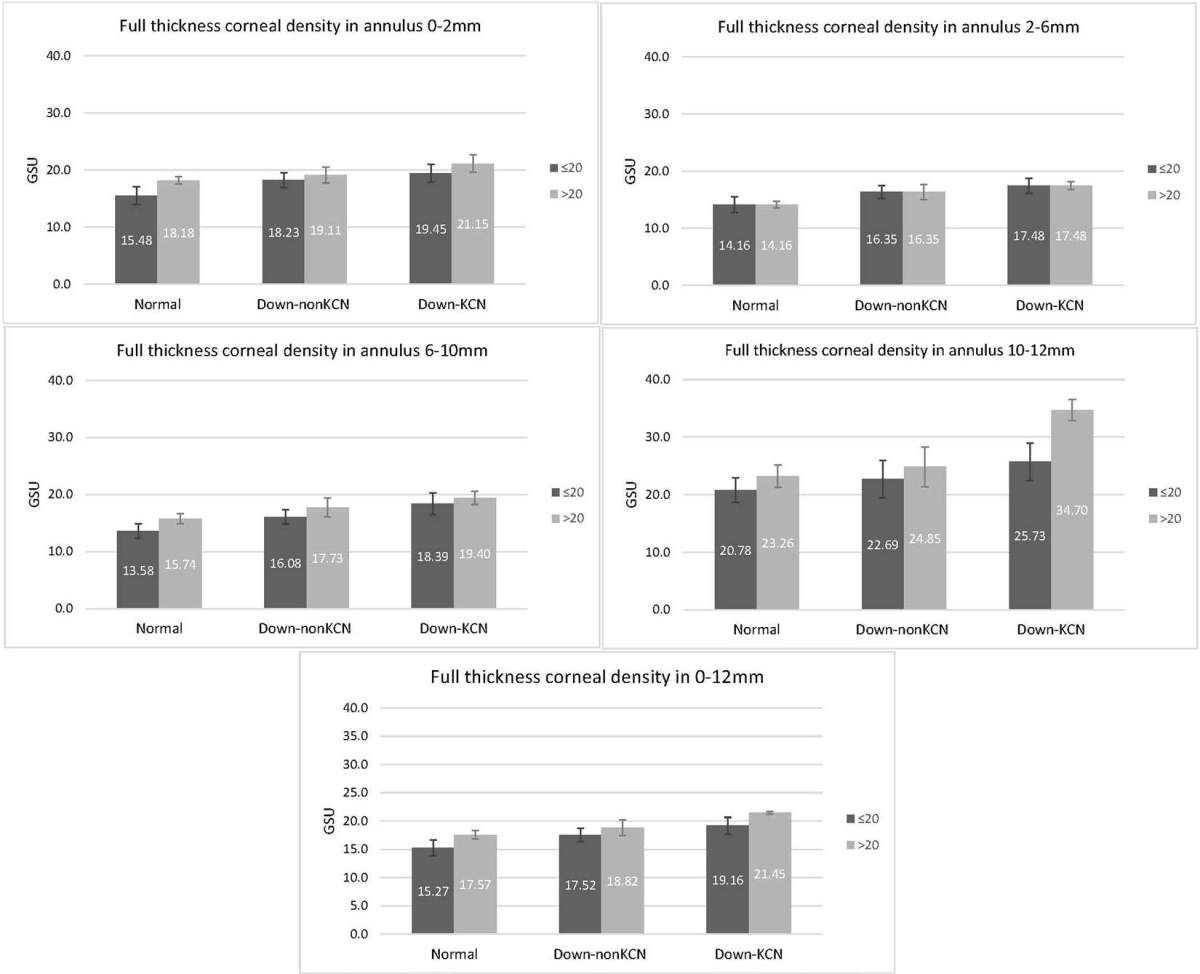
Full thickness corneal density (mean and standard deviation) in the 0–12 mm zone in Down patients with (Down-KCN) and without keratoconus (Down-nonKCN) and the normal age- and gender-matched control group compared in subgroups of age ≤20 and >20 years.

According to the multiple regression analysis, full thickness density in the 0–12 mm increased with age in Down-KCN (β = 0.16, P = 0.036), Down-nonKCN (β = 0.14, P < 0.001), and the normal control (β = 0.12, P = 0.006) groups. Figure 2 shows the full thickness density in different zones and annuli in two age subgroups (≤20 and >20 years) in the three groups. The 0–12 mm full thickness density directly correlated with the average 0–8 mm corneal thickness value in the Down-nonKCN (β = 0.01, P = 0.011) and normal control (β = 0.01, P = 0.016) groups. However, there were no significant correlations with gender (all P > 0.050) or corneal volume (all P > 0.050) in any of the three groups. In the other words, corneal density significantly correlated with age and corneal thickness, but not with corneal volume.

## Discussion

In the present study, we determined the corneal density in two groups of Down syndrome patients (Down-KCN and Down-nonKCN) and compared results with a group of normal (non-Down and non-KCN) age- and gender-matched individuals. To our knowledge, this is the first report concerning light backscatter in different corneal layers and zones in patients with Down syndrome. Owing to the groups included in the study, it was possible to evaluate the individual effect of Down syndrome and KCN on corneal density. The average repeatability for this index, as measured with Pentacam, is reported to be 3.3 ± 1.8%, which is affected by the poor measurement repeatability in peripheral regions^14^.

### Effect of KCN on Densitometry

Literature suggests that several factors, such as the size and arrangement of collagen fibrils, can influence corneal transparency and density^15^. Given the disarrangement of the corneal collagen structure in KCN patients, their corneal densitometry is expected to differ from normal subjects^16^. In this study, all middle layer indices were higher in the Down-KCN group than the Down-nonKCN patients. Despite numerous studies, there is still no consensus whether KCN is a disease of the anterior or posterior cornea. Some believe that KCN affects the anterior layers of the cornea^17,18^ and some consider KCN a posterior phenomenon^19,20^. In our Down-KCN group, since density readings in the middle layer were significantly different from Down-nonKCN in all zones and annuli, KCN appears to have affected the middle thickness layer of the cornea most. In the anterior and posterior thickness layers however, inter-group differences were only significant in the corneal periphery (beyond the 6 mm zone) where the repeatability of the measurements should also be taken into consideration. In a study of normal subjects^14^, the variability of full thickness densitometry readings in 6–10 mm and 10–12 mm annuli was reported 2.8% and 6.4%, respectively. In patients with Down syndrome, the repeatability of peripheral measurements may be even weaker due to specific eye morphology and a different corneal structure. Therefore, it can be said that the peripheral difference between Down-KCN and Down-nonKCN groups in all three thickness layers falls within the measurement repeatability of the indices, and the real difference between the two groups is in the middle thickness layer and up to the 6 mm zone. Therefore, in Down syndrome patients, this index (densitometry of the middle thickness layer of the central 6 mm zone) could be a more sensitive parameter for the diagnosis of early KCN when there is still no visible change in topographic, elevation, or pachymetric parameters^21^.

### Effect of Down Syndrome on Densitometry

In the present study, all indices were higher in the Down-nonKCN group compared to the normal control group up to the 10 mm diameter. Wang *et al*.^22^ showed that alterations in chromosome 21 can be associated with changes in corneal collagen fibers and consequently corneal biomechanics, which may explain the results of the present study that show increased corneal densitometry in patients with Down syndrome. Patients with Down syndrome, even in the absence of KCN, have thinner and steeper corneas as well^23,24^. A thin corneal stroma^25^ as well as hydrops^26^ in patients with Down syndrome can contribute to increased light scatter. Since in addition to the middle layer, the anterior and posterior layers showed different scatter in both Down syndrome groups (with and without KCN) compared to normal cases, further studies are needed to examine the changes in the anterior and posterior thickness layers and distinguish the impact on the epithelium and endothelium compared to the stroma.

The multiple analysis showed that total corneal densitometry increased with age which is in line with other studies^27,28^. This may be due to increased corneal opacity as a result of aging, and the increase has been reported to occur even in the 20- to 30-year age range^2^. Results also indicated that total corneal density directly correlated with age and corneal thickness, albeit to a weaker degree. This correlation was observed both in the Down-non-KCN and normal control groups, but not in the Down-KCN group which had a smaller sample size. Given the weak correlation coefficient between corneal density and thickness in the former groups (β = 0.01), the association may be attributed to the larger sample sizes of these two groups, and thus, considered clinically negligible. In other words, the association between corneal density and thickness is due to the effect of age, and corneal density is independent of corneal thickness and volume.

We found no relationship between gender and densitometry. Previous studies have been inconclusive on the association between gender and corneal transparency. For example, Olsen^27^ and Ni Dhubhghaill *et al*.^14^ found no relationship while Hillenaar *et al*.^28^ reported more scatter in men, and Garzon *et al*.^2^ reported more scatter in women (clinically insignificant). A comprehensive study can help us understand the role of inter-gender hormonal or chromosomal differences that may affect corneal density.

The limitation of this study was the small size of the Down-KCN group that may have affected the results in terms of inter-group differences. In conclusion, the findings of study suggest that Down syndrome is associated with increased density and light scatter in all corneal layers up to the 12 mm diameter, even in the absence of KCN. In the presence of KCN, however, there is increased density and light scatter in the middle layer within the central 6 mm zone with no additional impact on the density in the anterior or posterior thickness layers. In other words, in Down syndrome patients, increased corneal density is a characteristic of Down syndrome, however, a middle-layer corneal density >17.5 GSU in the 0–2 mm zone and >15.5 GSU in the 2–6 mm zone can be indicative of KCN. These results can help clinicians distinguish KCN in Down syndrome patients and avoid misdiagnoses and overestimations. Further studies are needed to understand when such changes occur compared to other signs and symptoms of KCN, and to determine the value of densitometry indices in identifying KCN cases.

## Methods

### Participants

This report is part of a comparative study conducted in 2016 at Noor Eye Hospital in Tehran. In this study, 250 patients with Down syndrome were recruited through the nation’s special needs schools, relevant non-profit organizations, and the Down Syndrome Society. The sampling details were mentioned in another paper^29^. Diagnosis of Down syndrome was based on the results of karyotype testing in their medical records. These patients underwent complete examinations by two independent subspecialists, and the diagnosis of KCN was made based on clinical examinations and tomographic data. The diagnostic criteria have been described previously^30^. In brief, a definitive diagnosis of KC was made when there were clinical signs and two or more abnormal topographic parameters. All suspected KCNs were followed for one year and no KCN signs was observed. The comparison group consisted of 200 normal (non-Down and non-KCN) samples referring to the Anterior Segment Clinic for refractive surgery consultation (initial preoperative evaluation) or a routine check-up. All these participants had normal corneas and underwent refractive surgery later.

### Eligibility criteria

The inclusion criterion for all three groups in this study was age between 10 and 30 years, and the exclusion criteria were the presence of pterygium or any history of ocular surgery. For Down syndrome cases, exclusion criterion was any concurrent disability including Klinefelter syndrome and autism. The exclusion criteria for the control group were any personal of family history of mental disability or having any signs of KCN.

### Ethical consideration

The study protocol was reviewed and approved by the Ethics Committee of Tehran University of Medical Sciences (ID: 1397–091) and adhered to the tenets of the Helsinki Declaration at all stages. All procedures were performed in accordance with the relevant guidelines and regulations of the Committee. The purpose and methods of the study were explained to the control group, and written informed consents were obtained. Since the law regards Down syndrome patient’s incompetent to give consent, all necessary information was discussed in the presence of their parents who provided written informed consents for their children’s participation in the study, and verbal assent was obtained from patients before each procedure.

### Measurements

All participants had complete vision tests and ophthalmic examinations. Clinical findings were insignificant, and there were no signs of inflammation or infection. Down syndrome cases had never used contact lenses, and participants in the control group had discontinued use at least three weeks prior to the exam. To avoid the effect of diurnal variations, ocular imaging with Pentacam HR (software version 6.08r27, data management version 1.21r24) was performed between 8:00 am and noon at least two hours after participants’ waking time. Imaging was repeated until the “Ok” quality status was obtained. In cases where imaging needed to be repeated more than three times, another appointment was made for 2–3 days later to avoid measurement error.

For measures of corneal density, expressed in GSU, a total of 20 indices were extracted. These included one densitometry reading from each of the 5 zones and annuli (0–2 mm, 2–6 mm, 6–10 mm, 10–12 mm, and 0–12 mm) in 3 different depth layers of the anterior 120 µm, posterior 60 µm, the middle layer, and the full thickness of the cornea. Corneal volume of the 10 mm diameter zone was also extracted and added to the database.

### Statistical analysis

Given the high correlation between fellow eyes (lowest = 0.890 for density in 0–12 mm zone of the middle layer and highest = 0.942 for the 0–2 mm annulus of the anterior layer), only right eye data were used in the analyses. The normality of the data distribution was tested by Q-Q plot. The three groups were compared using analysis of variance and post hoc tests. Multiple linear regression was used to investigate the concurrent effects of age, gender, and group variables (Down-KCN, Down-nonKCN, and control) on each index of corneal density. In this model, corneal density indices were entered as dependent variables, the group was entered as the independent variable (as a dummy), and age and gender were treated as confounders. Multiple linear regression was also used to assess the correlation between 0–12 mm full thickness corneal density and total volume with adjustments for age, gender, total corneal thickness (0–8 mm) in three study groups. Given the fact that corneal indices tend to change with age, comparisons were repeated with subgroups of ≤20 and >20 years of age. The significant level was considered 0.05.

## Data Availability

Data supporting the findings in this report are available from the corresponding author (HH) upon reasonable request.
